# Books

**Published:** 1882-09

**Authors:** 


### Books.

MENTAL PATHOLOGY AND THERAPEUTICS. By W. Griesinger, M,D. Translated from the Second German Edition, By C. Lockhart Robertson, M.D., Cantab., and James Rutherford, M.D., Edin. New York: William Wood & Company. 1882. Wood's Library of Standard Medical Authors.

The note appended to the volume by the translators is dated June, 1867. The first edition of the work appeared, so we are informed in the preface, in 1845. Hence it is not among the most recent productions upon this subject; but it need not necessarily be the worse for that circumstance.

The translators claim that their rendering has been very accurate and literal. Judging from the heaviness of style and the labored mode of expression which characterizes the book throughout, we should say that their claim was well founded, and that the German text had been closely followed.

The contents of the volume are divided into five books. The first is devoted to general considerations. Then follows, The cause and mode of origin of mental disease; forms of mental disease; the pathological anatomy of mental disease and the prognosis and treatment of mental disease. Insanity, says the writer, is to be divided according to the form and nature of the psychical anomalies, and, in the present state of our knowledge, its classification must of necessity be a purely symptomalogical one. He follows, therefore, in his description of the disorder, the classification of Esquirol, proposed in 1838—melancholia, monomania, mania, dementia and idiocy. The author very properly emphasizes the necessity for vigorous interference at the earliest possible period, at the very commencement of the malady, even before its full development, to prevent its establishment, if possible, and to prevent its becoming chronic if its advent cannot be wholly averted. It is at this stage usually that the general practitioner has it in his power, by prompt and proper treatment, to accomplish so much for this unhappy class of sufferers. The book contains 375 pages. It represents much valuable information for the medical man and bears evidence of very close application on the part of the author.

---

Lectures on Diseases of Children. A Handbook for Physicians and Students. By Dr. Edward Henoch, Professor in the University of Berlin. New York: William Wood & Co. 1882. Wood's Library of Standard Medical Authors.

The author tells us that this work contains almost exclusively the personal experience which he has had the opportunity of collecting during a practice of thirty-seven years, and his own experience has been made the basis not alone of the clinical descriptions, but also of the therapeutical recommendations, he, however, modestly adds, as a matter of course, the observations of any single physician will nevertheless remain imperfect. In speaking of the importance of appropriate nourishment for infants our author says: when mother's milk cannot be obtained, cow's milk is the only proper substitute, the excess of casein being compensated by sufficient dilution. Cow's milk is the best substitute for mother's milk during the entire period of infancy, and other substitutes should only be administered when good cow's milk cannot be obtained. He does not approve the use of condensed milk on account of the enormous amount of sugar—about sixty per cent. —which it contains, and which is liable to cause acid fermentation and diarrhœa.

Unfortunately, he goes a step further and seriously impairs confidence in the value of his advice by indorsing several brands of farinaceous preparations, called by manufacturers and commercial men ' infants' foods."

Under the head of pseudo-croup we find "salt pork constantly applied to the throat" recommended and emetics condemned! In discussing intestinal entozoa he says: "All possible convulsive diseases have been attributed to the reflex irritation of worms, but personally I have hardly ever been able to detect such a connection." Notwithstanding his "thirty seven years personal experience."

The paragraphs on treatment are, as a rule, brief and unsatisfactory, and the care bestowed upon the description of symptoms and of the morbid anatomy does not compensate this defect, as it is the object of the practical physician to cure his patients, not to make autopsies for the purpose of confirming his diagnoses.

The formulary, in the back of the volume, is rendered unavailable to many readers as it is expressed exclusively according to the metric system of weights and measures, which is a great mistake on the part of medical writers in the present state of public sentiment and public knowledge regarding the decimal system.

---

TRANSACTIONS OF THE MICHIGAN STATE MEDICAL SOCIETY. For the year 1882. George W. Topping, M.D., DeWitt, President, G. E. Ranney, M.D., Lansing, Secretary. Lansing: W. E. George & Co. 1882.

This volume of 184 pages, contains the proceedings of the seventeenth annual meeting of the society, the address of the retiring president, Dr. J. H. Jerome, Saginaw, on the objects and interests of the organization, besides the following papers: Two cases of mal-presentation by E. P. Christian, M. D. Insanity of masturbation, by C. B. Burr, M.D. Legal responsibility of surgeons for ununited fractures, by Foster Pratt, M.D. Remarks on the treatment of laryngeal phthisis, by E. L. Shirley, M.D. Suppurative catarrh of the middle ear, by Eugene Smith, M.D. Optic neuritis, by Leartus Connor, M.D. After treatment of tracheotomy, by Henry J. Reynolds, M D. Antisepsis in the treatment of disease, by D. W. C. Wade, M.D. Urinary deposits, by J. A. Post, M.D. Notes on uterine tumors, by G. E. Ranney, M.D.

